# dUTPase: the frequently overlooked enzyme encoded by many retroviruses

**DOI:** 10.1186/s12977-015-0198-9

**Published:** 2015-08-12

**Authors:** Amnon Hizi, Eytan Herzig

**Affiliations:** Department of Cell and Developmental Biology, Sackler School of Medicine, Tel Aviv University, 69978 Tel Aviv, Israel

**Keywords:** dUTPase, Retroviruses, Reverse transcription, dUTP, Mutagenesis, Beta-retroviruses, Non-primate and primate lentiviruses, HIV, Endogenous retroviruses

## Abstract

Retroviruses are among the best studied viruses in last decades due to their pivotal involvement in cellular processes and, most importantly, in causing human diseases, most notably—acquired immunodeficiency syndrome (AIDS) that is triggered by human immunodeficiency viruses types 1 and 2 (HIV-1 and HIV-2, respectively). Numerous studied were conducted to understand the involvement of the three cardinal retroviral enzymes, reverse transcriptase, integrase and protease, in the life cycle of the viruses. These studies have led to the development of many inhibitors of these enzymes as anti-retroviral specific drugs that are used for routine treatments of HIV/AIDS patients. Interestingly, a fourth virus-encoded enzyme, the deoxyuridine 5′-triphosphate nucleotidohydrolase (dUTPase) is also found in several major retroviral groups. The presence and the importance of this enzyme to the life cycle of retroviruses were usually overlooked by most retrovirologists, although the occurrence of dUTPases, particularly in beta-retroviruses and in non-primate retroviruses, is known for more than 20 years. Only more recently, retroviral dUTPases were brought into the limelight and were shown in several cases to be essential for viral replication. Therefore, it is likely that future studies on this enzyme will advance our knowledge to a level that will allow designing novel, specific and potent anti-dUTPase drugs that are effective in combating retroviral diseases. The aim of this review is to give concise background information on dUTPases in general and to summarize the most relevant data on retroviral dUTPases and their involvement in the replication processes and pathogenicity of the viruses, as well as in possibly-associated human diseases.

## Background

The hallmark of all retroviruses is their replication strategy that relies on two critical steps. The first is the reverse transcription of the viral plus strand RNA into linear double-stranded DNA that is catalyzed by the viral reverse transcriptase (RT). The second step occurs when the synthesized DNA is subsequently integrated by the viral integrase (IN) enzyme into the cell genomic DNA [1–4]. After integration, the proviral DNA becomes a part of the cellular genomic DNA. The genome of all retroviruses is organized in three major distinct genes: *gag*, *pol* and *env*. In most retroviruses, the *pol* gene encodes for all three basic retroviral enzymes, the protease (PR), RT and IN [1]. Due to the critical role of these three retroviral enzymes in the viral cycle, a massive body of research was conducted on them. These studies were combined with a very extensive search for drugs effective against HIV, the AIDS causing retrovirus. This was done mainly by searching for selective inhibitors of the viral PR, RT and IN enzymes. Indeed, almost all anti-HIV/AIDS drugs that are currently used to treat patients are inhibitors of these three enzymes. In combination anti-HIV/AIDS therapies, several inhibitors (sometimes against more than one viral enzyme) are administered. Remarkably, relatively little attention was given to a fourth enzyme, encoded by several groups of retroviruses, the deoxyuridine 5′-triphosphate nucleotidohydrolase (dUTPase, EC 3.6.1.23). This lack of attention results probably from the absence of dUTPases from those retroviruses that were the most heavily studied so far (such as, HIV and other primate lentiviruses, gamma-retroviruses or the alpha-retroviruses). Thus, even reviews on retroviruses usually overlook this enzyme.

**Fig. 1:**
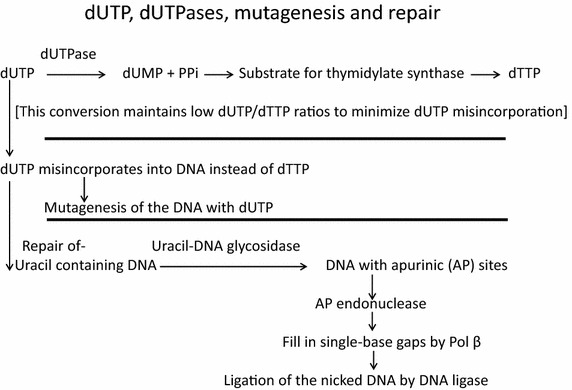
A schematic description of the pathways for the introduction of dUTP into DNA and the repair of uracilated DNA.

Cellular dUTPases hydrolyze dUTP into two products, dUMP and pyrophosphate (PPi). Subsequently, the product dUMP is used as a substrate for thymidylate synthase in the major biosynthesis pathway to dTTP. Therefore, dUTPases have essential roles in preserving low cellular dUTP over dTTP ratios [5–7]. Lowering the intracellular dUTP/dTTP ratios obstructs dUTP misincorporation into DNA, since most DNA polymerases can use dUTP instead of dTTP for DNA synthesis, a process that may result in introducing mutations into the synthesized DNA (Fig. 1). To keep uracil residues out of DNA, most organisms, both prokaryotes and eukaryotes, as well as several DNA viruses (e.g., herpesviruses and poxviruses) and some groups of retroviruses, encode dUTPases that can be essential for their viability [5, 8, 9]. In this review, we will describe the most relevant information regarding the presence and functions of dUTPases in retroviruses and their involvement in the life cycle of the viruses and of the infected cells.

## Review

### Retroviruses and the retroviral life cycle

Most retroviruses belong to the ortho-retrovirinae sub-family that is divided, according to genome organization, into two major groups, the simple retroviruses and the complex ones [1]. All retroviruses contain three principal coding domains with information for virion proteins: (1) *gag* that directs the synthesis of internal virion proteins that usually form the matrix and capsid structures. (2) *Pol* that encodes for the RT and IN enzymes. (3) *Env* that encodes for the surface and transmembrane glycoproteins of the viral envelope. An additional smaller domain that is present in all retroviruses is *pro* that encodes the viral protease. This *pro* gene can be part of the *gag* (as in the case of the alpha-retroviruses), as an independent gene (in the beta-retroviruses), or part of *pol* (as in gamma-retroviruses, lentiviruses or in delta-retroviruses) [1]. Simple retroviruses usually carry only this essential genomic information, whereas the complex retroviruses encode also for additional small regulatory proteins that are derived from multiply-spliced viral mRNAs.

The most unique process in the life cycle of retroviruses is the complex reverse transcription step that takes place in the cytoplasm of the virus-infected cells [3, 4, 10, 11]. After penetrating the target cells, the retroviral single-stranded plus-sense RNA, still contained within the viral core complex proteins, undergoes reverse transcription. In this critical step, the viral RNA is copied into a double-stranded DNA by a combination of the enzymatic activities of the core-associated RT enzyme. These activities are the DNA polymerase, capable of copying both RNA and DNA, and the ribonucleases H (RNase H) that hydrolyzes the RNA template in RNA–DNA heteroduplexes formed during reverse transcription. This multi-step process involves also two template switches (or strand transfers) that result in a duplication of sequences located at the 5′ and 3′ ends of the virion RNA, so they are eventually fused in tandem to both ends of the generated viral DNA, forming the long terminal repeats (LTRs). After completion of DNA synthesis, the resulting product, still in complex with viral proteins (now called pre-integration complex—PIC), is translocated into the nucleus and the DNA is integrated into the host cell DNA by the enzymatic activities of the viral IN [2, 10, 12]. This integrated provirus becomes part of the cellular genome and, after activation, is transcribed into the viral mRNAs. The unspliced full-length RNA can serve as the viral progeny RNA genome. In addition, this mRNA and the spliced mRNA species are used to synthesize the various viral proteins.

After infection with simple retroviruses, the transcription control is mediated primarily by interactions of cellular factors with the viral LTR, whereas in complex retroviruses, some viral regulatory proteins can affect transcription as well. Since most cells targeted by exogenous infective retroviruses can survive the infection, once integrated into germ cell genomes, the retroviral genomes can be transferred vertically in the infected animals for millions of years. These sequences of the endogenous retroviruses (ERVs), after being fixed in the evolutionary lineage, became integral part of most eukaryotic cells. Consequently, these endogenous viruses vastly outnumber the exogenous retroviruses [10, 13, 14]. Thus, human endogenous retroviruses (HERVs) constitute nearly 8 % of the human genome [15, 16]. Many of these sequences were fixed in the germ line of old-world monkeys after their evolutionary separation from new-world monkeys about 35 million years ago.

### dUTPases in general

Cellular dUTPases (EC 3.6.1.23) hydrolyze dUTP to dUMP and PPi, thus serving two essential functions in DNA metabolism (Fig. 1). First, the dUMP product is a primary substrate for thymidylate synthase in the major dTTP biosynthesis pathway. Second, dUTPases help to maintain low intracellular dUTP/dTTP ratios. This is necessary to minimize the misincorporation of uracil into DNA, since most DNA polymerases (except for some archaeal enzymes) cannot distinguish between thymine and uracil and the uracil/thymine incorporation ratio depends on the relative level of dUTP and dTTP. The misincorporation into the synthesized DNA of the non-canonical deoxyribo-nucleotide, dUTP, can eventually result in mutagenesis, for review, see [7, 9]. An excessive DNA repair of the uracilated DNA is initiated by uracil DNA glucosidases (UNG) that remove the uracil, forming an apurinic DNA [7–9]. This DNA is then repaired by a chain of enzymatic activities of apurinic endonuclease, DNA polymerase beta and DNA ligase (Fig. 1). UNGs have evolved in all organisms as the most common form of DNA-repair enzymes. Thus far, six groups of the UNG superfamily have been discovered and studied to varied extents [17]. However, under constant high dUTP/dTTP ratios, uracil residues will be incorporated again and again instead of thymidines during repair synthesis. This vicious circle of uracil re-incorporation and repair is likely to lead to an accumulation of many double strand breaks and strand exchanges that will eventually result in thymidine-less cell death [18].

Given the critical role of dUTPases in cell metabolism, it is not surprising that their presence was shown to be essential for the survival of both prokaryotic cells, such as *Escherichia coli* [19], and eukaryotic cells, such as *Saccharomyces cerevisiae* [20]. The enzyme was also identified in *Plasmodium falciparum*, *Mycobacterium tuberculosis*, trypanosome and human cells, as well as in DNA viruses and in several groups of retroviruses and even some bacteriophages (for an updated review—see [7]). Accordingly, inhibiting cellular dUTPase activities by drugs can impair cell growth. Therefore, this approach might be also applied for treating infections by specific pathogens. However, to attain selectivity against the pathogens (without debilitating the host cells), this strategy is particularly applicable for treating infections by protozoan organisms, as their dUTPases have evolved differently from bacteria and eukaryotic cells, thus forming a completely distinct family of proteins [21].

In many cases, from mammal to plant cells, cellular dUTPases were shown to be both development and cell cycle regulated, with elevated activity in undifferentiated dividing cells and low levels in terminally-differentiated and/or non-dividing cells [22–24]. Consequently, the levels of cellular dUTPase activity may parallel the size of the deoxynucleotide pool, which is high in dividing cells, such as activated lymphoblasts, and very low in non-dividing cells, such as macrophages [25]. In some eukaryotes, two dUTPase isozymes are generated by mRNA alternative splicing or by using alternative promoters. Thus, human cells contain nuclear and mitochondrial isoforms, where the nuclear one is under cell cycle control, while the mitochondrial isoform is constitutive [26].

Interestingly, recent reports that use different models have done away with the dogma that DNA uracilation is always deleterious. Thus, deamination of cytosine bases in DNA to uracil by the activation-induced deaminase is obligatory for the diversity of immunoglobulin genes [27, 28]. Moreover, rather than being dangerous, HIV DNA uracilation can benefit the early phase of the viral life cycle by inhibiting auto-integration [29]—see below. Finally, it was recently demonstrated that *Drosophila melanogaster* tolerates high levels of uracil in DNA during some developmental stages, suggesting a novel role of uracil-containing DNA in *Drosophila* [30]. Assuming that DNA uracilation is beneficial to some biological processes, and since cellular dUTPases are involved in this scenario, it may be that dUTPases can also perform other unrelated cellular functions. Indeed, a line of novel studies has shown that, apart from their pivotal role in lowering dUTP levels, cellular dUTPases play other roles in regulating several key cellular processes—by serving as signaling molecules in both prokaryotic and eukaryotic cells. Thus, dUTPases were shown to be involved in the transfer of mobile genetic elements that carry and disseminate virulence genes in prokaryotes and in the regulation of the immune system in autoimmunity and apoptosis. These unexpected dUTPase-associated “moonlighting” activities (defined as activities of catalytically-active proteins with divergent dual functions) open new research opportunities to explore the mechanisms, by which they serve as cellular regulators. Since these diverse regulatory functions of dUTPases were extensively reviewed recently [31], we will not elaborate here on these exciting and promising novel aspects of dUTPases. However, it should be noted that even in some DNA viruses, dUTPases affect host-cell interactions via mechanisms independent of dUTP hydrolysis. Recent studies have shown that the dUTPases, encoded by the gamma-herpesviruses, Epstein-Bar virus, Kaposi’s sarcoma-associated herpesvirus and MHV-68, control the immune system. This is done by various mechanisms, such as, upregulating cytokines, activating NF-kB pathway, targeting cytokine receptors or down-regulating ligands recognized by NK cells (thus, controlling NK attacks) and inhibiting type I interferon signaling pathway [32–34]. dUTPases of other human herpesviruses were found to modulate dendritic cell function and innate immunity [35]. Moreover, some of the data show that enzymatically-inactive dUTPases can also perform these regulatory functions, suggesting that their biological roles are not merely associated with their dUTPase enzymatic activity. Further studies are required to explain how these functions evolved to be associated with dUTPase enzymes. Likewise, new interesting evidence, regarding the potential involvements of the dUTPases of HERVs in human diseases, also alludes to possible cellular regulations that are independent of catalytic activity of dUTPase [36, 37], see below.

### Structure and catalysis of dUTPases

**Fig. 2:**
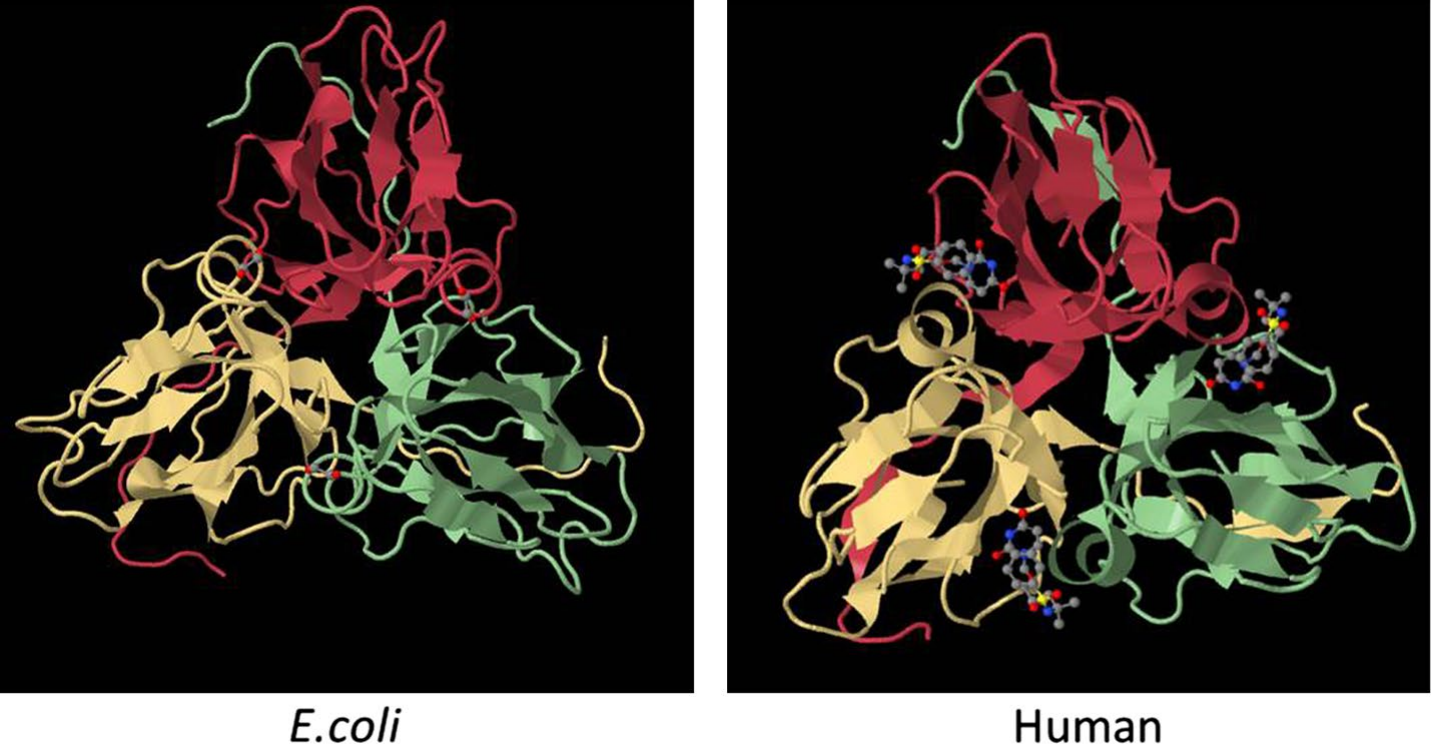
The three-dimensional structures of human and bacterial dUTPases. The homo-trimeric *E. coli* (2HRM) and Human (3ARN) dUTPases were illustrated using the Jsmol internal viewer (http://www.PDB.org). Structures are presented in the front C3 axis orientation, with each subunit in a different color. Ligands (methylene-dUTP in the *E. coli* enzyme and dUTPase inhibitor in the Human counterpart) are presented in stick configuration.

While the gamma-phosphate group of nucleoside triphosphates is relatively reactive (as in ATP or GTP), the alpha-phosphate position, as well as the phosphodiester bonds in nucleic acid, are significantly more inert, thus help prevent aberrant modifications [38]. Reactions at these sites require powerful enzyme catalysts, such as nucleases, polymerases [39] and dUTPases [7]. Almost all studied dUTPases, including those of bacteria, human and retroviruses, are homo-trimeric proteins [7]. The homo-trimers form three identical active sites in a symmetric fashion—see Fig. 2. In contrast, herpesviruses [40, 41] and *Caenorhabditis elegans* [42] dUTPases are distinct as monomers. Here, trimer**-**mimicking monomers are formed from a genome that encodes all three enzyme monomers, within the same gene (with linker regions located among the subunits). A third occasion was reported for protozoan dUTPases that function as dimers, but contain none of the five conserved sequence motifs typical of the dUTPases of the former two groups. Thus, they have probably evolved differently from dUTPases in bacteria and other eukaryotes [21, 43].

In general, homo-trimeric or trimer-mimicking dUTPases, including the retroviral ones, are characterized by a series of five conserved amino acid motifs [7] (see also below). Among mammalian viruses, this common set of motifs was initially identified by sequence comparisons in herpesviruses, retroviruses and poxviruses. Thought initially thought to be “pseudo-protease”, comparisons of these motifs with the *E. coli* dUTPase sequence and other known dUTPases revealed their identity as unique dUTPase motifs [5, 44]. The importance of these conserved motifs for the catalytic function was established by mutagenesis and by X-ray crystallography of a variety of dUTPases. To prevent wasteful and undesired hydrolysis of energy-rich NTPs or dNTPs, dUTPases must be highly specific to their related substrate. This is provided by two major mechanisms, reviewed in [7]. In short, the first one is a steric exclusion of purines, thymine and ribose and the second is hydrogen bonding that is specific to uracil. The steric hindrance is mediated by residues from motif 3, which form a tight beta-hairpin that binds uracils and deoxyribose and exclude thymine and purines. Altogether, the specificity of dUTP binding by dUTPases is provided by the uridine moiety that fits precisely into the enzyme’s active site [7]. The dUTPase binding pockets, present in each of the three subunits are highly specific for uracil. Phosphate chain coordination involves magnesium ions and is analogous to that in DNA polymerases. Due to conformational changes in the enzyme during catalysis, most crystal structures have not resolved the residues in the C-terminus. All homo-trimeric dUTPases share similar mechanisms that lead to efficient catalysis. In several crystal structures, a water molecule is positioned for a nucleophilic attack on the dUTP alpha-phosphate that leads to phosphate ester hydrolysis. This attack is coordinated by the side chain of the highly conserved aspartate that is located in motif 3 [45, 46]. Interestingly, in herpesvirus dUTPases, though the conserved motifs are preserved, they are arranged in a different manner. Yet, these motifs still fold similar to usual trimeric dUTPases [7, 41].

### The dUTPase-encoding genes in retroviruses

**Fig. 3:**
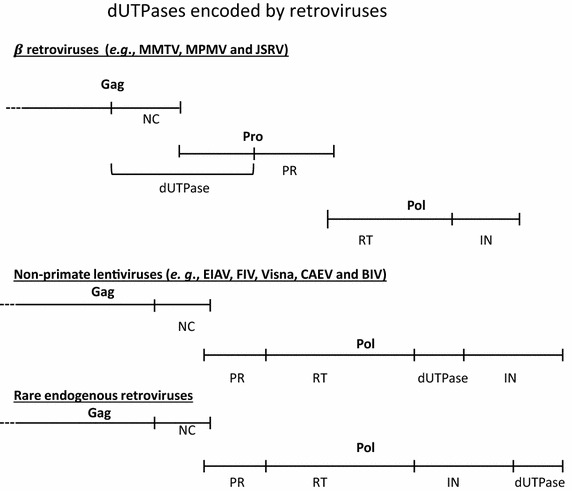
The biogenesis of dUTPase-expressing retroviruses. This schematic description of the various precursor polyproteins, encompassing the dUTPase proteins, reflects also the position of the dUTPase-encoding genes in the different retroviral groups (described in detail in the text). These schemes are not drawn to scale.

Despite their variable positions, dUTPase-encoding genes in many dUTPase-encoding organisms were observed to be adjacent to other genes that are involved in nucleotide metabolism, such as ribonucleotide reductase, transcription initiation factors, primase, and DNA synthesis flavoprotein [44]. A similar pattern of gene localization also exists in retroviral dUTPases, where, in most cases, the dUTPase-encoding genes are located in the same genome segment encoding for the viral RT, IN and or PR proteins. In addition, in the case of beta-retroviruses the nucleic acids binding protein (NC) is an integral part of the viral dUTPase (see Fig. 3).

Unlike most other retroviral enzymes, in the dUTPase-expressing viruses, the encoding gene is situated in two different genomic locations, suggesting two diverse evolution pathways (Fig. 3). In the beta-retroviruses, the *gag*-*pol* genes have three reading frames (*gag*, *pro* and *pol*) [1] and the dUTPase protein is encoded by both the *gag* and *pro* reading frames [47–51]. Consequently, the dUTPase protein is actually a trans-frame polypeptide. To be more specific, the dUTPase N-terminal segment (of about 90 residues) is derived from the C-terminus of the Gag polyprotein. Therefore, this segment is identical to the entire viral nucleocapsid (NC) protein [52] (also known in these viruses as p14). The C-terminus of the dUTPase is encodes by the 5′ portion of the *pro* gene, ending adjacent to the PR-encoding segment. In these viruses, the total length of the dUTPase is about 240 amino acids residues and it is a proteolytic product of the Gag-PR polyprotein precursor. This fused Gag-PR polyprotein results from ribosomal frameshifting that occurs during translation (see [53]). In contrast, the *gag*-*pol* genes of lentiviruses have only two reading frames. In the dUTPase-expressing lentiviruses, the encoding gene is located within the *pol* gene, between the RT’s RNase H (C-terminal) and the IN-encoding parts. Thus, dUTPase is a proteolytic product of the Gag–Pol polyprotein precursor. Interestingly, the polypeptide length of the majority of the lentivirus-associated dUTPases is about half (~130 residues) of that of the beta-retroviral dUTPases. A rare exception to the pattern in non-primate lentiviruses was reported for scarcely studied endogenous retroviruses (ERVs) [54–56]. Here, the dUTPase gene is located also within the *pol* gene, but rather at its 3′ terminus; hence, it is C-terminal to the IN (Fig. 3).

Since all reoviruses have very small genomes, any genetic information included in these genomes must be vital to the viruses. Therefore, even with little knowledge about the importance of virus-coded dUTPases, it is highly likely that they are essential for replication. Despite sharing similar mechanisms of replication, only several groups of retroviruses encode dUTPases, while others lack this enzyme. The major dUTPase-expressing retroviruses are the beta-retroviruses and the non-primate lentiviruses, whereas most other viral groups (including primate retroviruses) lack the enzyme. It might be that this difference in the requirements for a viral-encoded enzyme depends on the cells infected by the viruses. Thus, alpha or gamma-retroviruses that lack a viral dUTPase replicate mainly in dividing cells, which have high endogenous dUTPase levels, while the dUTPase-expressing retroviruses can infect also non-dividing cells with low dUTPase activity (vide supra). In several retroviruses, early DNA sequence comparison analyses have suggested some resemblance of unidentified genes to the viral protease; hence, they were initially termed protease-like domains or pseudo-proteases [57]. Only later on, the homology to the dUTPase gene was confirmed [5]. The key study by Elder and associates has subsequently confirmed the presence of a catalytic dUTPase activity in particles of several retroviruses [58].

In the dUTPase-expressing retroviruses, the location of encoding gene can affect the level of the protein’s expression. The *gag* portion of the *gag*–*pol* polycistronic mRNA is translated approximately 20 times more than the entire polycistron that has to undergo one or two −1 nucleotide frameshifting events to complete translation [1, 53]. Thus, in the beta-retroviruses, where the N-terminal part of dUTPase is derived from Gag, there is a higher level of dUTP expression compared to non-primate lentiviruses. Indeed, such a high expression enabled one of us to detect the first retroviral dUTPase-related protein in virions of mouse mammary tumor virus (MMTV), already in 1987 [49]. Relatively large amounts of the protein (designated at the time as p30, due to its ~30 kDa size) were isolated from virions and analyzed by protein sequencing. The data showed that this protein is a trans-frame protein, as its N-terminal sequence was identical to the viral NC, and its C-terminus was derived from the N-terminal half of Pro. Only later on, after a study on the presence of dUTPases in retroviral virions was published [58], this MMTV p30 was expressed as a recombinant protein and shown to possess a dUTPase catalytic activity [48, 51].

**Fig. 4:**
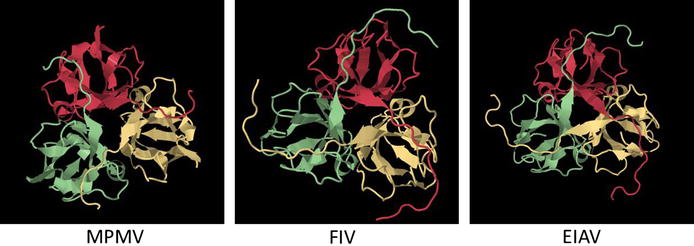
The three dimensional structure of three representative retroviral dUTPases. The structures of the homo-trimeric dUTPases of MPMV (3TRL), FIV (1F7D) and EIAV (1DUN) were illustrated employing the Jsmol internal viewer (http://www.PDB.org). The structures are presented in the back C3 axis orientation with each subunit in a *different color.*

**Fig. 5:**
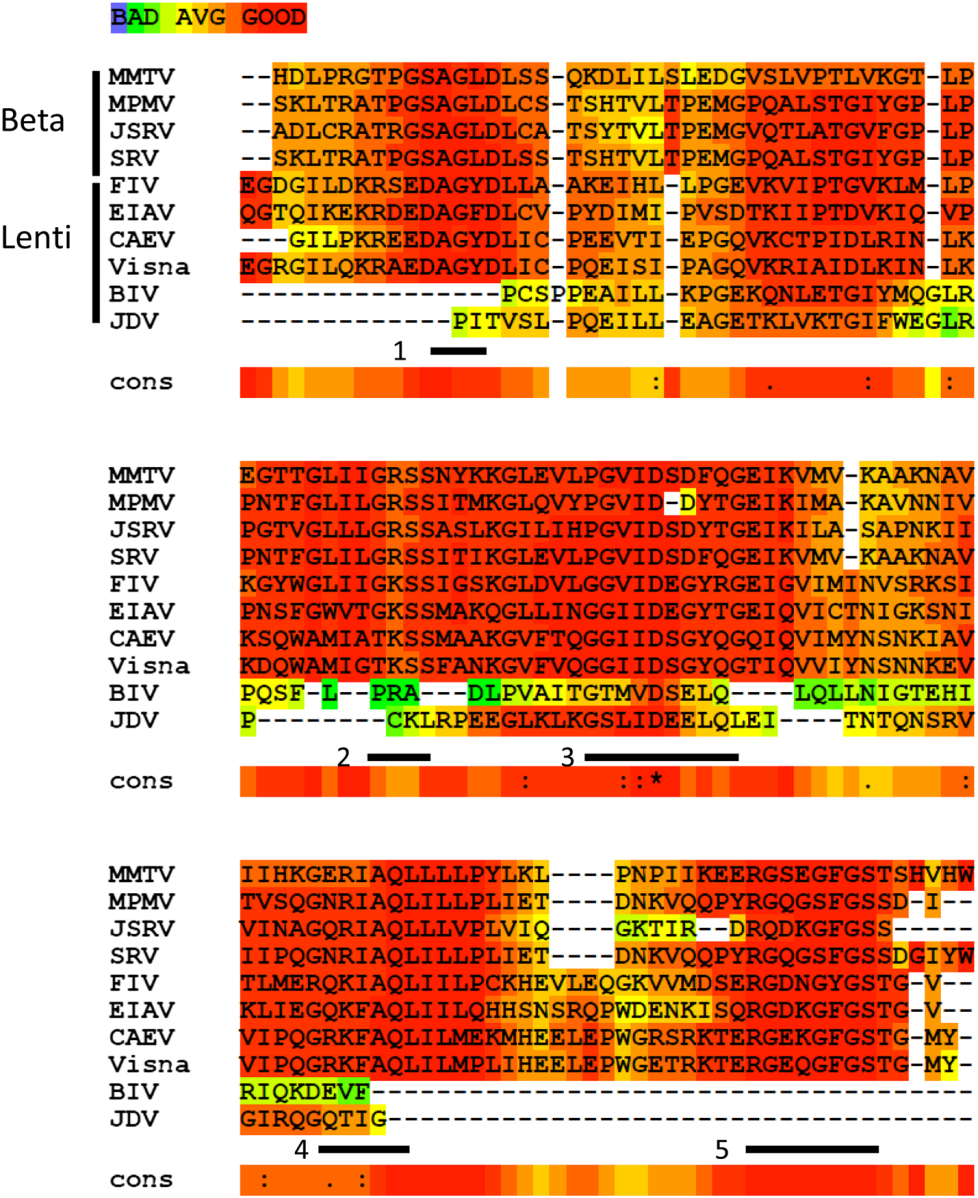
Multiple sequence alignment of the retroviral dUTPases. The sequences of dUTPases in the major exogenous retroviruses described in the text were analyzed. The five highly-conserved domains typical of dUTPases are indicated below the sequences. In the case of the beta retroviruses, the NC-derived N-terminal sequences were not included in the alignment. The sequences of the following dUTPases are as follows: EIAV (GI: 157830894); FIV (GI: 1942421); Visna (GI:9626549); BIV (GI: 9626219); CAEV (GI: 266706151); JDV (GI:733067);MMTV (GI: :9626965);MPMV (GI: 9627210);SRV (GI: 334748);JSRV (GI: 9626914). The figure was prepared using the T-COFFEE alignment tool.

**Fig. 6:**
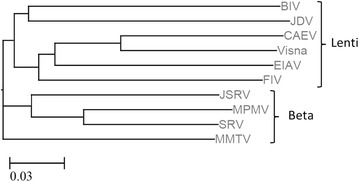
Phylogenetic tree of dUTPase sequences from exogenous infective retroviruses. This phylogenetic tree was constructed using amino acid multiple alignments and the neighbor-joining method with ETE-toolkit (http://etetoolkit.org/treeview/), with the sequences shown in Fig. 5. *Scale bar* represents the *p*-distance (the observed number of nucleotide differences per site).

Like most dUTPases, all studied retroviral dUTPases are homo-trimeric in their three-dimensional structure (Fig. 4). Accordingly, enzymatically active retroviral dUTPases possess the five conserved domains, typical to all homo-trimeric or trimer-mimicking dUTPases, Fig. 5 [7]. Some families of endogenous retroviruses, notably, HERVs K and ERVs share also dUTPase-related motifs with the five conserved segments [13, 59, 60]—see below. In contrast, the putative dUTPases of the non-primate lentivirus, bovine immunodeficiency virus (BIV) was recently shown by us to have a sequence with only a partial resemblance to these conserved motifs with no detectable enzymatic activity [61]—see below. The phylogenetic tree of most exogenous retroviral dUTPases, discussed below, is shown in Fig. 6. It is highly likely that after their separation throughout evolution, the beta-retroviral dUTPase-encoding gene has evolved separately than that of the non-primate lentiviruses. For more general phylogenetic trees that include also dUTPases from endogenous retroviruses, see [13, 60].

### Beta-retroviruses

All beta-retroviral dUTPases are bi-functional due to their chimeric nature. Here, the viral Gag-derived NC proteins are fused at their C-termini to the Pro-derived dUTPase conserved domains. The dUTPase proteins from two prototype viruses of this group, MMTV and Mason Pfizer monkey virus (MPMV), were the mostly studied. The information pertaining to dUTPases from other viruses in this group, Jaagsiekte sheep retrovirus (JSRV) and simian retrovirus (SRV), was mainly based on sequence homology to the more studied dUTPases [47, 62], see Fig. 5. In all beta-retroviruses, the NC is 81–95 residues long and the Pro-derived segment is 153–154 residues, out of a total of ~240 residues long polypeptide [63]. This means that the NC sequence can encompass more than a third of the whole enzymatically-active dUTPase protein. Retroviral NC has a variety of activities that are central to viral replication, as it has a nucleic acid chaperoning activity through its conserved basic residues and zinc-finger structures, for a review—see [52]. This chaperone function, in conjunction with the protein’s aggregating function, is up-modulated by successive NC processing events, resulting in the condensation of the viral NC. Reverse transcription also depends on NC processing. Inducing NC dissociation from double-stranded DNA leads to the formation of the PIC that is capable of host chromosomal integration. In addition, NC interacts with cellular proteins, some of which are involved in viral budding, and also with several viral proteins. This collection of activities is likely to substantially affect the mature beta-retroviral dUTPases (that were shown always to retain the NC). Indeed, three retroviral proteins, IN, capsid and NC, were found to be capable of physical interaction with MPMV dUTPase [64]. This protein is present in stable form that resists proteolysis by retroviral and cellular proteases in virions as well as in virus-infected cells. MPMV dUTPase retains both nucleic acid binding and dUTP hydrolyzing catalytic activity. Sequence comparison of beta-retroviral dUTPases with other dUTPases reveals that the beta-retroviral enzymes have evolved to support the proper function of the NC protein fused to their dUTPase domain. This evolution affected the whole sequence, except for the five conserved motifs (see Fig. 5), as shown by the relatively low sequence similarity (<30 %) between beta-retroviral and other dUTPases [63]. The modifications, however, do not provoke changes in the protein’s overall fold. Yet, due to the basic nature of the NC segment, the beta-retroviral dUTPases have a much higher, basic pI, compared with the more acidic isoelectric points observed in most dUTPases [7, 63]. The catalytic rate constant of the recombinant protein is, however, about tenfold lower than in other dUTPases (including those from lentiviruses) [47]. In the case of retroviral dUTPases, this feature may compensate for the higher levels of beta-retroviral dUTPase expression, relative to lentiviral dUTPases (see above). In all, it may be that the coupling, within a single protein subunit, of the nucleic acids binding function with dUTPase activity, could facilitate the attachment of the dUTPase to sites, where DNA synthesis by RT takes place, thus hydrolyzing in situ the incoming dUTP.

Enzyme kinetics of recombinant MPMV dUTPase and a truncated protein segment (without the NC domain) suggested that the NC domain has no adverse effects on enzymatic activity and that oligonucleotide binding to the NC domain may modulate enzymatic activity [47]. These results failed to provide an explanation for the ~tenfold lower catalytic rate constant. A shorter linker region located between conserved motifs 4 and 5 was observed in all beta-retroviral dUTPases relative to other dUTPases (including a four-residue deletion relative to the non-primate lentiviral dUTPases)—see Fig. 5. This may suggest that the missing connecting residues can cause a steric constraint that lowers the k_cat_ value. High-resolution X-ray structures, combined with modeling, indicate that the fusion with NC domains alters the conformation of the flexible C-terminus by disturbing the orientation of a critical beta-strand. Accordingly, this segment is capable of double backing upon the active site of its own monomer and is stabilized by non-covalent interactions formed with the NC terminal segment. In this case, the homo-trimeric dUTPase fold is modulated in a specific manner that allows the accommodation of the additional NC segment. Such a co-folding of the dUTPase terminal segments, which was not observed in other dUTPases, results from the presence of the fused NC domain [63]. Elaborate studies on MPMV dUTPases were conducted to show the mechanism of the alpha attack-mediated dUTP hydrolysis that is carried out by the enzyme [64]. Here, a combination of diverse structural methods, as well as the knowledge of other dUTPases, unveiled molecular details of the catalytic nucleophilic attack and identified novel enzyme-product intermediates.

Although the dUTPase of MMTV was identified and characterized before the MPMV counterpart was investigated [48, 49, 51], substantially fewer studies were performed on the MMTV enzyme. A comparative study of the dUTPases of MMTV, herpes simplex and *E. coli* showed that the two viral enzymes are less specific to dUTP than the bacterial one [65]. The MMTV enzyme has a reduced discrimination against dTTP and UTP, while it is still selective against dCTP.

Despite the relatively large body of research conducted on beta-retroviral dUTPases, we could not find any studies on the biological importance of the enzyme to the life cycle and infectivity of the viruses. However, this missing information can be complemented by the studies described below on involvement of dUTPases in the infectivity of the non-primate lentiviruses.

### Lentiviruses

#### Non-primate lentiviruses

Among all exogenous lentiviruses, dUTPase-encoding genes were observed in only the non-primate lentiviruses. However, there are traits of these genes in some ERVs, including in many HERVs (see below). Among the non-primate lentiviruses, dUTPase-encoding genes are present in feline immunodeficiency virus (FIV) [66], puma lentivirus [67], equine infectious anemia virus (EIAV) [68, 69], caprine arthritis-encephalitis virus (CAEV) [70] and visna virus of sheep [71]. Additionally, in the bovine lentiviruses, BIV that is associated with a debilitating cattle disease [72], and Jembrana disease virus (JDV), a homologous dUTPase-encoding gene is present [73, 74], see above (Fig. 5). In a rare case of the infectious small-ruminant genotype E lentivirus (isolated from goats and sheep), almost the entire dUTPase genome is deleted [75]. As mentioned above, the dUTPase encoding gene in this retroviral group is part of the *pol* gene and is situated between the RT and IN encoding segments (Fig. 3). Another feature that sets the non-primate lentiviral dUTPases apart from the beta-retroviral counterparts (and from other known dUTPases as well) is the relatively shorter polypeptide subunit, of about 130 residues (which is roughly half of the beta-retroviral dUTPases). Despite this major difference, similar to other studied dUTPases, the three-dimensional structure of EIAV dUTPase exhibits a homo-trimeric arrangement, where each subunit folds into a twisted antiparallel beta-barrel with the N and C-terminal portions interacting with the adjacent subunits [76]. A generally similar structure was reported also for FIV dUTPase [77], see also Fig. 4.

The majority of the biochemical studies on the dUTPases of the non-primate lentiviruses were conducted on recombinant EIAV dUTPase. This enzyme was shown to be highly specific to dUTP and sensitive to inhibition by dUDP, with little inhibition by other nucleotides or the reaction products, dUMP and PPi [78]. In this study, mutational analyses were also performed by targeting a conserved domain present at the C-terminus of all dUTPases. This domain shares high homology with the phosphate binding loops (P-loops) of several ATP and GTP-binding phosphatases. The P-loop-like motif of dUTPases is glycine rich, but lacks the invariant lysine found in authentic P-loops. Deletion of this motif led to a loss of the enzymatic activity. In addition, a series of point mutations in EIAV dUTPase that inactivate these P-loops also abolished the dUTPase activity; thus establishing the importance of these loops for catalysis. Another study compared EIAV dUTPase with the *E. coli* counterpart [79]. The results showed that the viral enzyme was as potent as the bacterial one in hydrolyzing dUTP, albeit less specific. The inhibition of the EIAV enzyme by dTTP, dUMP and a synthetic analog is stronger by one order of magnitude than that of the bacterial counterpart. Transient kinetics of EIAV dUTPase showed that the rate constants for the association and dissociation of substrate and inhibitors were consistent with a one-step substrate binding mechanism [80]. After the flexible C-terminal part of the protein was removed by a limited proteolysis, the dUTPase activity was totally quenched, although substrate binding was hardly affected. This suggests that this terminus is indispensable for catalysis but not a for substrate binding.

Most studies on the effects of dUTPases on the biology of non-primate lentiviruses concluded that this enzyme is critical for replication only in non-dividing cells (such as primary macrophages). In contrast, dUTPase-defective viruses can grow quite well in dividing cells, where the dUTPase activity is supplied by the infected cell. This result is consistent with the data presented above, showing that cellular dUTPases are cell cycle regulated, with an elevated activity in dividing cells and low levels in terminally differentiated non-dividing cells. This finding regarding the non-primate lentiviruses was reported for the dUTPase of CAEV and visna [70], EIAV [68, 81, 82] and FIV [66]. The effects of viral dUTPase in virus-infected animals were also evident. Thus, in FIV-infected cats, virus burden was reduced due to dUTPase impairment, particularly in tissues, such as spleen and salivary gland [83]. The viral RNA load in plasma of Shetland ponies, infected with a dUTPase-defective EIAV, was 10 to 100-fold lower than in animals infected with the wild-type virus [68]. In the case of CAEV, the dUTPase is necessary for the development of bilateral arthritis lesions in the carpus of infected goats [84]. However, this is not always the case. Thus, visna virus dUTPase was found to be dispensable for neuro-pathogenicity [71]. Likewise, the dUTPase gene of FIV is not essential for neuro-pathogenesis in cats [85].

As expected, the importance of the dUTPase to the retroviruses is linked to the misincorporation of dUTP instead of dTTP. Several studies have linked the lack of dUTPase activity with an increased incidence of mutations in the viral DNA, especially G to A substitutions when the viruses replicate in terminally differentiated non-dividing cells. This result was found in CAEV [84], EIAV [81] and FIV [83]. These findings indicate that uracil accumulation in the viral DNA can be detrimental to the viral life cycle, although the precise mechanism it still not fully understood. HIV-1 RT was shown to introduce G to A mutations in a simple in vitro DNA synthesis, using highly-biased dNTP concentrations [86]. This can explain why many of the spontaneous mutations found in HIV-1 are G to A. As mentioned above, the levels of cellular dUTPase activity may parallel the size of the deoxynucleotide pool, which is high in dividing cells, such as activated lymphoblasts, and very low in non-dividing cells, such as macrophages [25]. Therefore, it is possible that in these dNTP-deficient cells, there are dU misincorporations across the template G, due to low dCTP concentrations, high dUTP (due to the lack of dUTPase) and the relative stability of dU-G mispairs, These conditions will eventually result in selective G to A transitions [84]. Apparently, despite the numerous studies conducted on dUTP misincorporation, this hypothesis still calls for further experimental support.

*The distinct case of BIV* The putative dUTPase genes of BIV and JDV are distinct, though both follow the pattern of non-primate lentiviruses [87, 88]. As in all non-primate lentiviruses, these genes are located between the RT and IN-encoding genes. However, in both cases the encoded polypeptide is substantially shorter than the ~130-residues protein of other lentiviruses, as it is only about 74 residues long [61, 73, 74]—see Fig. 5. This truncated polypeptide lacks extensive parts of the five conserved motifs, characteristic of the homo-trimeric dUTPases, or the whole motifs [61]. As far as we know, no other dUTPase-related protein, including all viral, prokaryotic or eukaryotic enzymes is so small. Still, it is highly likely that this dUTPase-related peptide has an important biological role, since it is conserved in both BIV and JDV [87, 88]. Our recent study showed that recombinant wild-type BIV dUTPase and infectious wild-type BIV virions were both dUTPase-defective, as no detectable enzymatic activity could be shown [61]. To assess the importance of the dUTPase gene to BIV replication, we generated virions of wild-type BIV or BIV with mutations in this gene. The two mutant dUTPases were the double mutant, D48E/N57S (located in the putative dUTPase active site and its vicinity) and a 36 residues deletion. Both mutant viruses were defective, as no progeny viruses were generated. Surprisingly, the cells infected with the mutant virions carry in their genomic DNA levels of integrated BIV DNA that were as high as in wild-type BIV-infected cells. This result shows that the dUTPase-mutated BIV strains could infect cells, as viral cDNA was synthesized and integrated. Yet, no new virions were generated from the infected cells [61]. Interestingly, all experiments were conducted in dividing cells, where, as mentioned above, the endogenous cellular dUTPase levels are supposed to be high. Therefore, according to this prediction, there should be only a minor effect of mutating the dUTPase-encoding gene, even in the case of an enzymatically-active dUTPase activity (let alone in the present case, where no activity was noticeable).

To explain these puzzling results, we speculated that either the integrated cDNA of the BIV mutants is defective (due to potential multiple mutations introduced during reverse-transcription) or that dUTPase mutations led to blocks in viral replication at steps post integration. These suppositions may implicate the involvement of BIV’s dUTPase in processes other than dUTP hydrolysis, thus highlighting its importance to BIV replication, despite the lack of any detectable catalytic activity. At this stage, several unexplored alternatives are open. For example, it might be that the viral protein interacts with cellular proteins (like UNG-see above) or it participates in late (post integration) stages of the retroviral replication cycle. Likewise, given the evidence on novel “moonlighting” activities of dUTPases, see above, such activities may be associated with the dUTPase of BIV. The information listed below on the possible involvement of HERV-K dUTPases with human diseases can also support this possibility. In any case, further studies are being performed now by us to answer the raised questions.

#### Human and primate lentiviruses

Primate exogenous lentiviruses (such as HIV-1, HIV-2 and simian immunodeficiency virus-SIV) evolved differently than other lentiviruses and are devoid of a dUTPase-encoding gene. Yet, one study proposed a weak albeit significant sequence similarity between HIV-1 gp120 envelop protein and human dUTPase [89]. This information may suggest that an ancestral dUTPase gene has evolved into the present CD4 receptor interacting region of gp120. Since these primate viruses can replicate also in non-dividing cells, where the dUTP/dTTP ratio is high (due to low cellular dUTPases, see above), it is likely that they can utilize other equivalent means to counteract the emergence of uracilated viral DNA genomes. Indeed, as an alternative, they can recruit one of the cellular UNG enzymes (UNG2) that are involved in the base-excision repair pathway (see above and Fig. 1). In HIV-1, there are conflicting reports about the identity of the viral protein that is responsible for this recruitment. Some studies implicated the viral IN [90–92], whereas others the accessory protein, Vpr [8, 93, 94]. Interestingly, the HIV-1 associated UNG2 could be replaced by packaging into the virions a heterologous dUTPase from CAEV. This finding suggests that UNG2 can counteract the dUTP misincorporation that results from the lack of the dUTPase [95]. A recent study established the essential steps through which UNG2 initiates the degradation of HIV-1 cDNA containing misincorporated dUTP and prevents viral integration [96].

Remarkably, there is another entirely independent mechanism to form uracil-containing DNA in many lentiviruses that eventually leads also to G to A mutations. This process is mediated by incorporating the cellular restriction proteins, APOBEC cytosine deaminases into Vif-deficient virions. This eventually leads to the impairment of virus replication due to C to U deamination of the synthesized viral cDNA. The APOBEC cytosine deaminase activity is largely specific to single-stranded DNA substrates and requires a minimum of five contiguous deoxy-nucleotides (three on the 5′ side of the target cytosine and one base on the 3′ side of the target cytosine). In wild-type viruses, the viral Vif protein recruits cellular CBF-β to form an E3 ubiquitin ligase complex that usually leads the APOBEC degradation. Due to its cardinal importance to HIV infectivity, this innate cellular anti-viral activity was heavily investigated, as part of the intensive investigations of cellular factors that restrict HIV-1 (for comprehensive reviews—see [97–100]). Therefore, we will not elaborate here on this process.

Taken together, as in the case of the other retroviruses, it is believed that uracilation of the viral cDNA is detrimental to the retroviral life cycle. This conclusion was also supported by in vitro evidence showing that the incorporation of uracils into minus-strand DNA during HIV-1 reverse transcription affects the specificity of plus strand synthesis initiation [101]. An interesting revision to the belief that uracilation has negative consequences was proposed by showing that HIV-1 could tolerate, or even benefits, from non-mutagenic uracil incorporation during reverse transcription [29]. Here, uracilation of the viral cDNA obstructs the strand transfer of the DNA ends that is catalyzed by the viral IN, thereby inhibiting the suicidal auto-integration side pathway and facilitating the correct chromosomal integration of viral cDNA and, consequently, the viral replication.

### Endogenous human retroviruses

About 8 % of the human genome comprise HERVs that represent fossilized sequences of ancient exogenous retroviruses [15], see also above. These elements, distributed in about 400,000 loci and transmitted vertically in a Mendelian manner, are classified into 30–40 families. Each family can encompass up to thousands of loci [102]. HERVs were suggested to be associated with a variety of human diseases, including, autoimmune diseases, neurological disorders and multiple malignancies, as well as involvement in placentation [16, 103]. Several HERV families were reported to harbor dUTPase domains. A sequence survey of various HERV families for the presence of dUTPase has found that ancestors of all HERV-K families but one encode dUTPases [60]. This phylogenetic analysis shows a monophyletic origin for the different HERV-K dUTPases. Sequences of the consensus dUTPase domains suggest that the various exogenous ancestors of HERV-K once encoded active enzymes. Interestingly, a recombinantly-expressed dUTPase was catalytically-active when a consensus sequence was constructed from independent genomic clones of HERV-K. This presumably-ancestral wild type HERV-K dUTPases was meticulously studied employing biochemical, mutagenic and structural approaches [59]. Despite this study, there are no available convincing reports that show the intracellular expression of HERV-encoded and catalytically active dUTPases. Even so, it was speculated, with no experimental proof, that HIV can lack a dUTPase-encoding gene, because the host human cells, infected by this exogenous virus, already express an endogenous dUTPase that is encoded by a HERV [104].

### Possible links of HERVs dUTPases to human diseases

As mentioned, the high presence of HERVs in numerous sites over the human genes has suggested their potential linkages to human diseases. Interestingly, stimulating new evidence suggest an unexplored specific linkage between the HERV-K encoded dUTPase and human psoriasis. Psoriasis is a chronic inflammatory immune disease of the skin that is characterized by an elaborate interplay between multiple-risk genes and their interactions with environmental factors. The psoriasis susceptibility locus 1 (PSORS1) mutation resided within a region close to human leukocyte antigen-C, designated risk haplotype (RH) 1/2, which is located within marker M6S168. This target region harbors fragments of a HERV-K. Two single nucleotide polymorphisms with alleles differing between high and low-risk haplotypes are located within the HERV-K dUTPase [105]. One of these haplotypes encodes a non-conserved Glu to Arg mutation. The HERV-K dUTPase is expressed in peripheral blood and in normal as well as in lesional psoriatic skin, thus suggesting that it can be a candidate gene for the PSORS1 mutation. To investigate the direct role of the HERV-K dUTPase in psoriasis, purified recombinant dUTPase versions with the wild-type sequence, and with mutations, reflecting the genotype characteristics of high and low-risk haplotypes, were evaluated to see whether they could modulate innate and/or adaptive immune responses [36]. The outcomes show that both wild-type type and mutant HERV-K dUTPase proteins induced NF-kB activation through Toll-like receptor 2 that is independent of the enzymatic activity. In both cases, the treatment of human primary cells with the recombinant dUTPase proteins triggered a secretion of TH1 and TH17 cytokines that are involved in forming psoriatic plaques, including IL-23, IL-12p40, IL-17, tumor necrosis factor-alpha, IL-8, and CCL20. This result was observed in dendritic/Langerhans-like cells and, to a lesser extent, in keratinocytes. An independent study described a variant discovery and case–control association of HERV-K dUTPase variants in 708 psoriasis cases and 349 healthy controls. Five common HERV-K dUTPase variants exhibited a high association with psoriasis, with the strongest association with a missense single-nucleotide polymorphism that leads to a K158R mutation. Haplotype analysis revealed that HERV-K haplotypes with the non-risk alleles significantly reduced the risk of psoriasis [37]. Moreover, functional testing showed higher antibody responses against recombinant HERV-K dUTPase in psoriasis patients compared with controls, as well as higher T cell responses against a single HERV-K dUTPase peptide.

The described studies support a novel and independent role for the HERV-K dUTPase in the susceptibility to psoriasis. Nevertheless, the mechanisms underlying the linkage between the HERV-K dUTPase and psoriasis are still elusive, especially since it does not require an enzymatically-active protein. In this respect, there is a partial similarity to our recent study on effects of the enzymatically-inactive BIV dUTPase [61], see also above. It is likely that, as described earlier for several outstanding cases [31], the HERV-K protein serves as a signaling molecule that is involved in affecting basic cellular functions. Apparently, the most exciting question is how the retroviral enzyme evolved to function in these “moonlighting” activities. It is expected that further detailed phylogenetically studies on retroviral dUTPases (similar to those already performed [13, 60]), in combination with intensive cell biology, will uncover new answers for this fundamental question.

## Conclusions

The data summarized in this review describe the importance of dUTPases to the retroviral life cycle in conjunction with cellular dUTPases, dUTP levels and mutagenesis (caused by dUTP misincorporation into DNA during the process of reverse transcription). Since both host cells and the infecting retroviruses have common goals to prevent and/or correct the detrimental effects caused by high dUTP levels, they have mutually developed analogous strategies to accomplish this objective. Interestingly, only several groups of retroviruses have evolved to express their own dUTPase, while others either take advantage of the cellular dUTPase or recruit a cellular UNG that initiates the correction of dUTP misincorporation. The association of HERV-K dUTPase with human psoriasis adds another twist to these relatively straightforward lines of reasoning, by highlighting the protein’s involvement in cellular signaling processes, which is not related to the catalytic dUTPase activity. The questions surrounding the function of the unique dUTPase of BIV lead also to new, yet unexplored directions. Taken together, there are still many research avenues that should be undertaken to better understand the diverse molecular mechanisms associated with retroviral dUTPases. Conceivable, the development of specific inhibitors of retroviral dUTPase activity, and/or dUTPase-interacting molecules, could greatly help in answering some of the raised questions and possibly help in designing novel anti-retroviral drugs.
