# Erratum to: dUTPase: the frequently overlooked enzyme encoded by many retroviruses

**DOI:** 10.1186/s12977-015-0202-4

**Published:** 2015-09-14

**Authors:** Amnon Hizi, Eytan Herzig

**Affiliations:** Department of Cell and Developmental Biology, Sackler School of Medicine, Tel Aviv University, 69978 Tel Aviv, Israel

## Erratum to: Retrovirology (2015) 12:70 DOI 10.1186/s12977-015-0198-9

The original version of this article unfortunately contained a typographical mistake. The spelling of ‘retroviruses’ was incorrect. The corrected text is given below.

Since all retroviruses have very small genomes, any genetic information included in these genomes must be vital to the viruses.

